# Supplementary material for: Deciphering spatial domains from spatially resolved transcriptomics with an adaptive graph attention auto-encoder
Source: Nat Commun. 2022 Apr 1;13:1739. doi: 10.1038/s41467-022-29439-6 (PMC8976049; doi:10.1038/s41467-022-29439-6)
Supplement: Supplementary file 1 — Supplementary Information [file 41467_2022_29439_MOESM1_ESM.pdf]

Supplementary Materials for

Deciphering spatial domains from spatially resolved transcriptomics with an adaptive graph attention auto-encoder

Kangning Dong[1,2] and Shihua Zhang[1,2,3,4*]

[1]NCMIS, CEMS, RCSDS, Academy of Mathematics and Systems Science, Chinese Academy of Sciences, Beijing 100190, China;
[2]School of Mathematical Sciences, University of Chinese Academy of Sciences, Beijing 100049, China;
[3]Center for Excellence in Animal Evolution and Genetics, Chinese Academy of Sciences, Kunming 650223, China;
[4]Key Laboratory of Systems Biology, Hangzhou Institute for Advanced Study, University of Chinese Academy of Sciences, Chinese Academy of Sciences, Hangzhou 310024, China.

*To whom correspondence should be addressed. Tel/Fax: +86 01 82541360; Email: zsh@amss.ac.cn.

Supplementary Notes

Comparison with other spatial domain identification methods

We compared STAGATE with the non-spatial clustering method implemented by SCANPY[1], and five recently developed spatial clustering approaches including Giotto[2], BayesSpace[3], stLearn[4], SpaGCN[5] and SEDR[6]. The parameter settings of these methods are as follows:

- **SCANPY:** The data preprocessing of SCANPY is the same as those of STAGATE (log-normalized and selecting the top 3,000 HVGs). The first 30 PCs were calculated and then the nearest neighbor network was constructed using the *scanpy.pp.neighbor()* function with default parameters. Finally, SCANPY obtains the clustering assignments using the *scanpy.tl.louvain()* function. For the DLPFC dataset）, the resolution parameter was tuned manually to ensure the number of clustering is equal to the ground truth.
- **Giotto:** The expressions was normalized according to parameter scalefactor=6000. The spatial network was first construct using the *createSpatialNetwork()* function with k=5 and maximum_distance_knn=1000. Then, the spatial domains were identified using *doHMRF()* function with parameter betas=c(0, 10, 20).
- **BayesSpace:** BayesSpace was applied to the DLPFC dataset according to its recommended parameters in the package vignette. Specifically, the input is the top 15 PCs of the log-normalized expression of the top 2,000 HVGs. The nrep parameter was set as 50,000 and the gamma parameter was set as 3. We only ran it for 10x Visium data because its package did not support the calculation of Slide-seq data[7, 8], and Stereo-seq data[9] directly.
- **stLearn:** We adopted stLearn on the DLPFC dataset using its recommended parameters in the package vignette. Specifically, the *stLearn.SME.SME_normalized()* function was performed on the raw counts of all genes with the parameter use_data="raw" and weights=" physical_distance". Then the first 30 PCs of the SME normalized matrix were used for further clustering and visualization. We only ran it for 10x Visium data because its package did not support the calculation of Slide-seq data and Stereo-seq data directly.
- **SpaGCN:** SpaGCN was applied to the DLPFC dataset according to its recommended parameters in the package vignette.
- **SEDR:** SEDR is the only method that can be applied to Slide-seq and Stereo-seq data among three existing spatial clustering methods. We ran SEDR for all experiments with its recommended parameters in the online tutorial (run_SEDR_DLPFC_all_data.py). Specifically, the parameter *k* is set as 10, and the epoch is set as 200.

STAGATE imputes gene expressions while preserving spatial expression patterns

We compared the imputation performance of STAGATE with four widely used single-cell RNA-seq imputation algorithms: MAGIC[10], DeepImpute[11], scVI[12], and DCA[13] in terms of Spearman correlation coefficients in terms of genes and Pearson correlation coefficients of the imputation values to the reference data. We collected publicly available ST data from similar tissue to construct three datasets, and conducted downsampling experiments in each section **(Supplementary Table S2**. Notably, STAGATE achieved the overall best performance on the DLPFC dataset and the Hippocampus dataset, and ranked second only to DeepImpute in the MouseBrain dataset (**Supplementary Fig. S14a, b**). Furthermore, compared to other imputation methods designed for single-cell RNA-seq, the usage of spatial information enables STAGATE to preserve the spatial expression patterns. STAGATE significantly improved the Moran's I values of spatially variable genes identified by SPARK-X compared to other approaches, which indicated that STAGATE enhanced their spatial expression signals (**Supplementary Fig. S14c**). We further calculated the z-score of the Moran's I value of the identified spatially variable genes, and found that STAGATE and scVI are the two best methods for preserving spatial patterns (**Supplementary Fig. S14d**). Notably, DeepImpute ranked first in the MouseBrain dataset, which included seven sections from the whole mouse brain, but performed poorly in the other two datasets. This result indicated that the similarity-based on expressions alone is not enough to perform accuracy imputation in the tissues with homogeneous cell types, such as the cortex and hippocampus. We also employed two examples to illustrate the ability of STAGATE to preserve spatial expression patterns. First, we visualized the raw expression and the imputed expression of the *KRT17* gene, which is enriched in cortical layer 6, in the DLPFC section 151676 (**Supplementary Fig. S14e**). Only STAGATE well revealed its spatial expression pattern. By contrast, MAGIC and DCA incorrectly imputed missing values in the white matter region, and scVI imputed those in both layers 1 and 6. Moreover, the expression of *Ociad2* showed strong specific expressions in the "cord-like" structure of the hippocampus in the raw data profiled by Slide-seqV2, and only STAGATE maintained its differential expression (**Supplementary Fig. S14f**).

Generating reference and downsampled datasets

For benchmarking imputation performance, we first selected highly expressed genes from the raw data of each section as reference expressions. We then performed synthetic downsampling simulations by randomly flip 30% non-zero entries of the reference to zeros. The specific criterion for gene filtering is to keep about 2,000 genes with the highest proportion of non-zero expressing cells. Imputation methods were implemented using default parameters on the raw data containing entire genes, and the measurements of imputation accuracy were calculated based on selected genes.

Measuring spatial autocorrelation using Moran's I statistic

The Moran's I statistic[14] is a measure of spatial autocorrelation measure, and can be used to rank genes by their spatially informative rank[15]. For each individual gene *i*, the Moran's I statistic is computed using the formula:

$$I = \frac{N}{S_0} \frac{\sum_{i=1}^{N}\sum_{j=1}^{N} w_{ij}\left(y_i - \bar{y}\right)\left(y_j - \bar{y}\right)}{\sum_{i=1}^{N}\left(y_i - \bar{y}\right)^2},$$

where y_i and y_j is the expression profiles of spot *i* and spot *j*, $\bar{y}$ is the mean expression of gene, *N* is the number of spots, w_{ij} is the distance weight between spot *i* and spot *j*, and S_0 is the sum of w_{ij}. Obviously, the Moran's I statistic is bounded between -1 (a chess board like pattern) to 1 (a clear spatial pattern). We used the implementation of *Seurat* package[16], and w_{ij} is set as 1/distance.

Selection of hyperparameter α

To explore the influence of the weight of cell type-aware SNN, we calculated the clustering assignments and UMAP plots of STAGATE under different α values **(Supplementary Fig. S20)**. We found that the clustering assignments were consistent when α=0.25 or 0.5. With the increase of α, the separation of tissue structures in UMAP plots was improved. However, when α=0.75 or 1, the performance of spatial domain identifications was impaired. For example, STAGATE with α=1 failed to identify the CA1sp domain within the hippocampus. Based on the above results, we guess that the results of STAGATE are robust under a small α, and a big α may reduce the flexibility of the graph attention mechanism. Thus, we set α=0.5 by default.

Supplementary Figures

Fig. S1. Comparison of spatial domains by clustering assignments via STAGATE, BayesSpace, SEDR, SCANPY, and manual annotation in the DLPFC dataset.
Fig. S2. Comparison of clustering accuracy on the DLPFC dataset.
Fig. S3. UMAP visualization and PAGA graphs generated by STAGATE, SEDR, stLearn, and SCANPY embeddings respectively.
Fig. S4. Comparison of spatial domains by applying Louvain clustering with different resolutions to the SCANPY, SEDR, STAGATE embeddings respectively in the Slide-seqV2 hippocampus data.
Fig. S5. Comparison of the UMAP visualization of SCANPY, SEDR, and STAGATE in the Slide-seqV2 hippocampus data.
Fig. S6. Visualizations of spatial domains identified by applying Louvain clustering to the STAGATE embedding and expressions of the corresponding marker genes in the Slide-seqV2 data of the mouse hippocampus tissue**.**
Fig. S7. Comparison of spatial domains by applying Louvain clustering with different resolutions to the SCANPY, SEDR, and STAGATE embeddings respectively in the Stereo-seq mouse olfactory bulb tissue section.
Fig. S8. Visualizations of spatial domains identified by applying Louvain clustering with resolution=0.8 to the STAGATE embedding and expressions of the corresponding marker genes in the Stereo-seq mouse olfactory bulb tissue section.
Fig. S9. Comparison of spatial domains identified by applying Louvain clustering with different resolutions to the SCANPY, SEDR, and STAGATE embeddings respectively on the Slide-seqV2 data of mouse olfactory bulb tissue section.
Fig. S10. Spatial trajectory inference in the Slide-seqV2 data mouse olfactory bulb tissues.
Fig. S11. Comparison of spatial domains identified by different methods in the adult mouse brain section profiled by 10x Visium.
Fig. S12. STAGATE reveals spatial domains in adult mouse posterior brain section profiled by 10x Visium.
Fig. S13. Comparison of spatial expression patterns before and after STAGATE denoising.
Fig. S14. Evaluation of imputation efficiency by downsampling the raw data.
Fig. S15. Spatial domains identified by applying mclust clustering to the SCANPY embedding in the 3D hippocampus model.
Fig. S16. Spatial domains detected in the mouse visual cortex STARmap data.
Fig. S17. Running time and GPU memory usage.
Fig. S18. Comparison of the spatial domain-specific expression patterns of STAGATE and SPARK-X.
Fig. S19. Statistics of the number of neighbors per spot in SNNs.
Fig. S20. Spatial domains and UMAP visualization generated by STAGATE under different α values.

Supplementary Tables

Table S1. Comparison between current bioinformatics methods for spatial domain identification of ST data.

Table S2. Description of all ST datasets used in this study.

Table S3. Description of Slide-seq data used to reconstruct the 3D hippocampus model.

Table S4. Sample IDs and URLs of data downloaded from Allen Brain Atlas in this study.

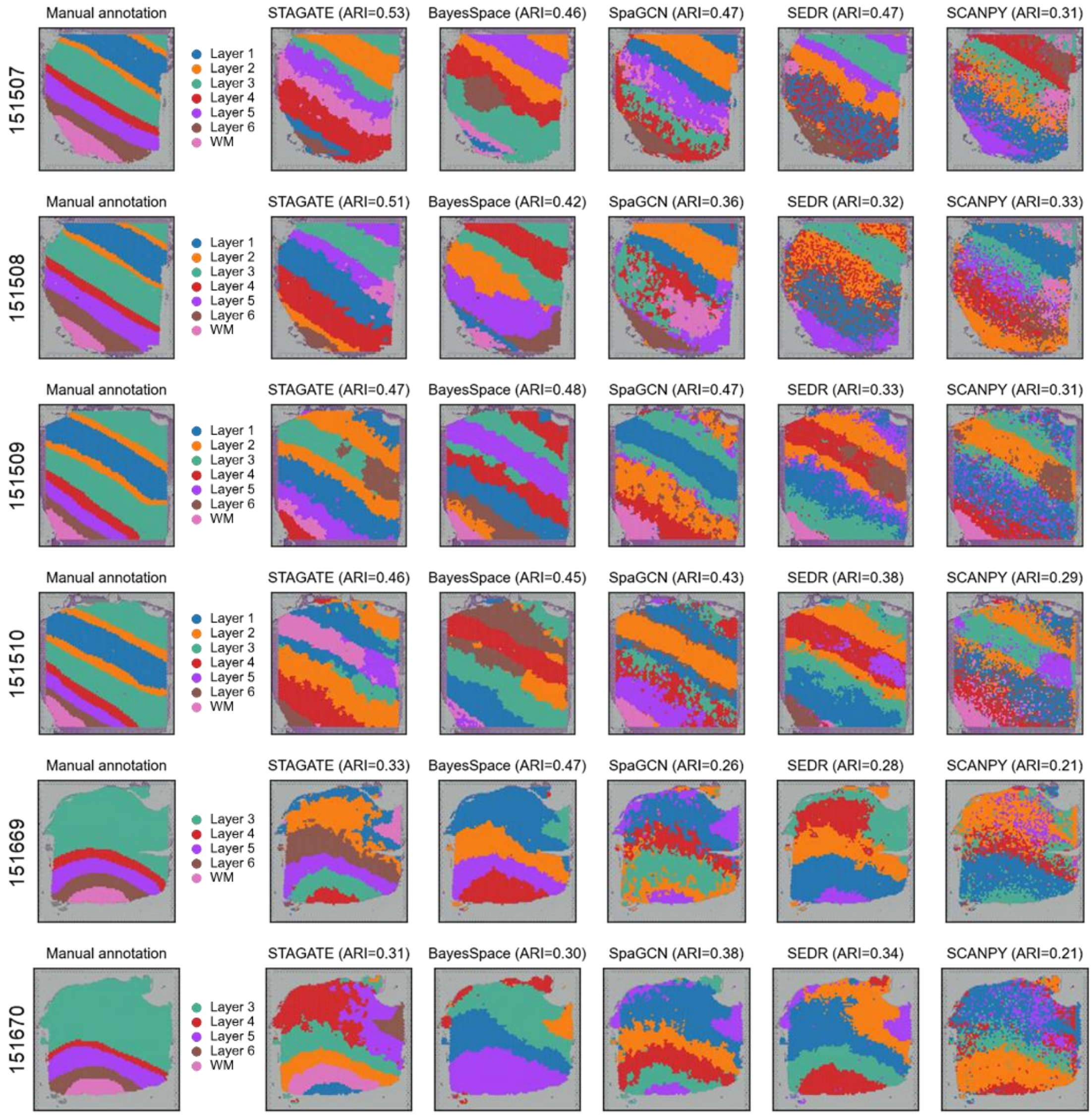

See next page

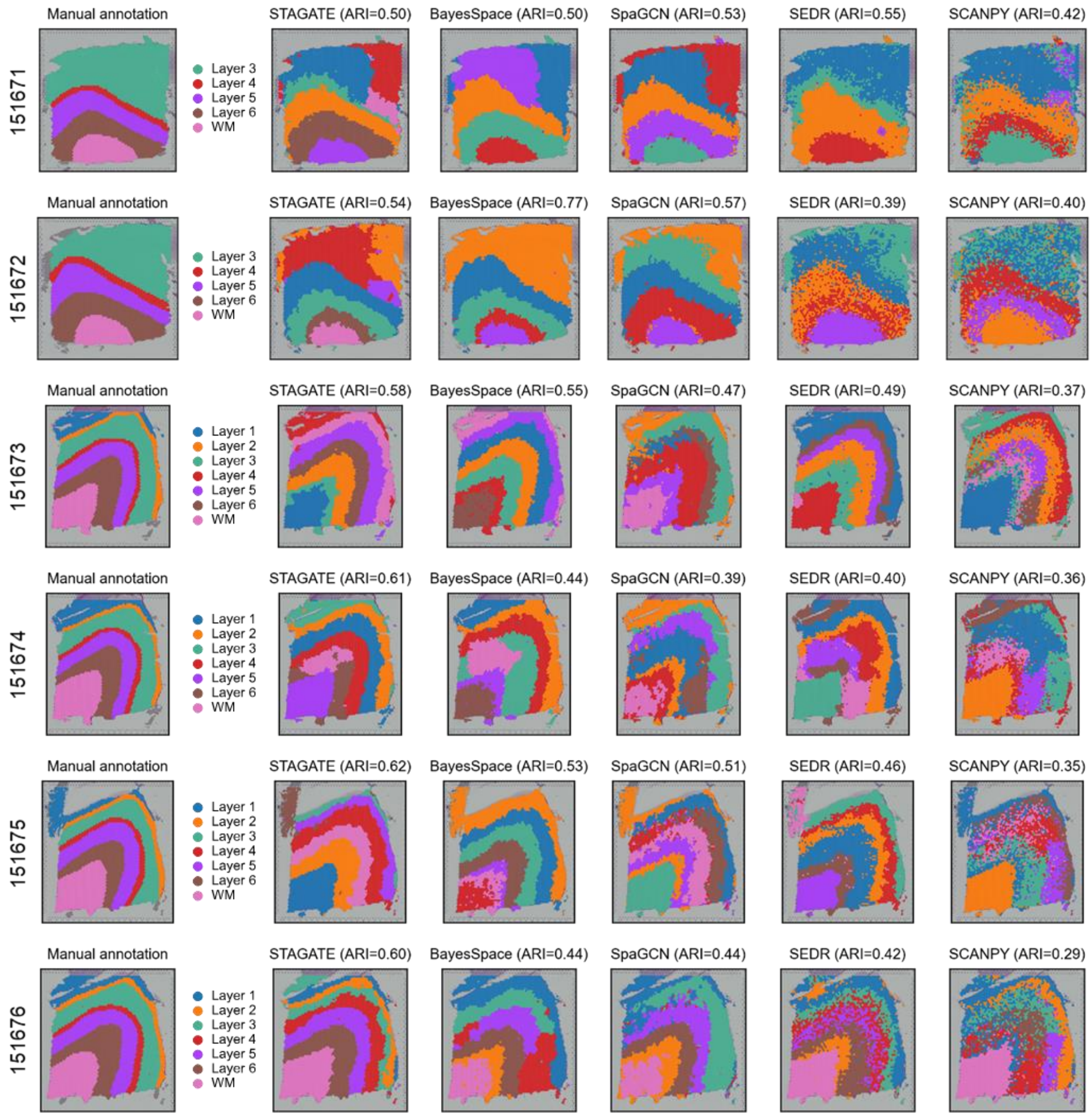

Fig. S1. Comparison of spatial domains by clustering assignments via STAGATE, BayesSpace, SEDR, SCANPY, and manual annotation in all 12 sections of the DLPFC dataset.

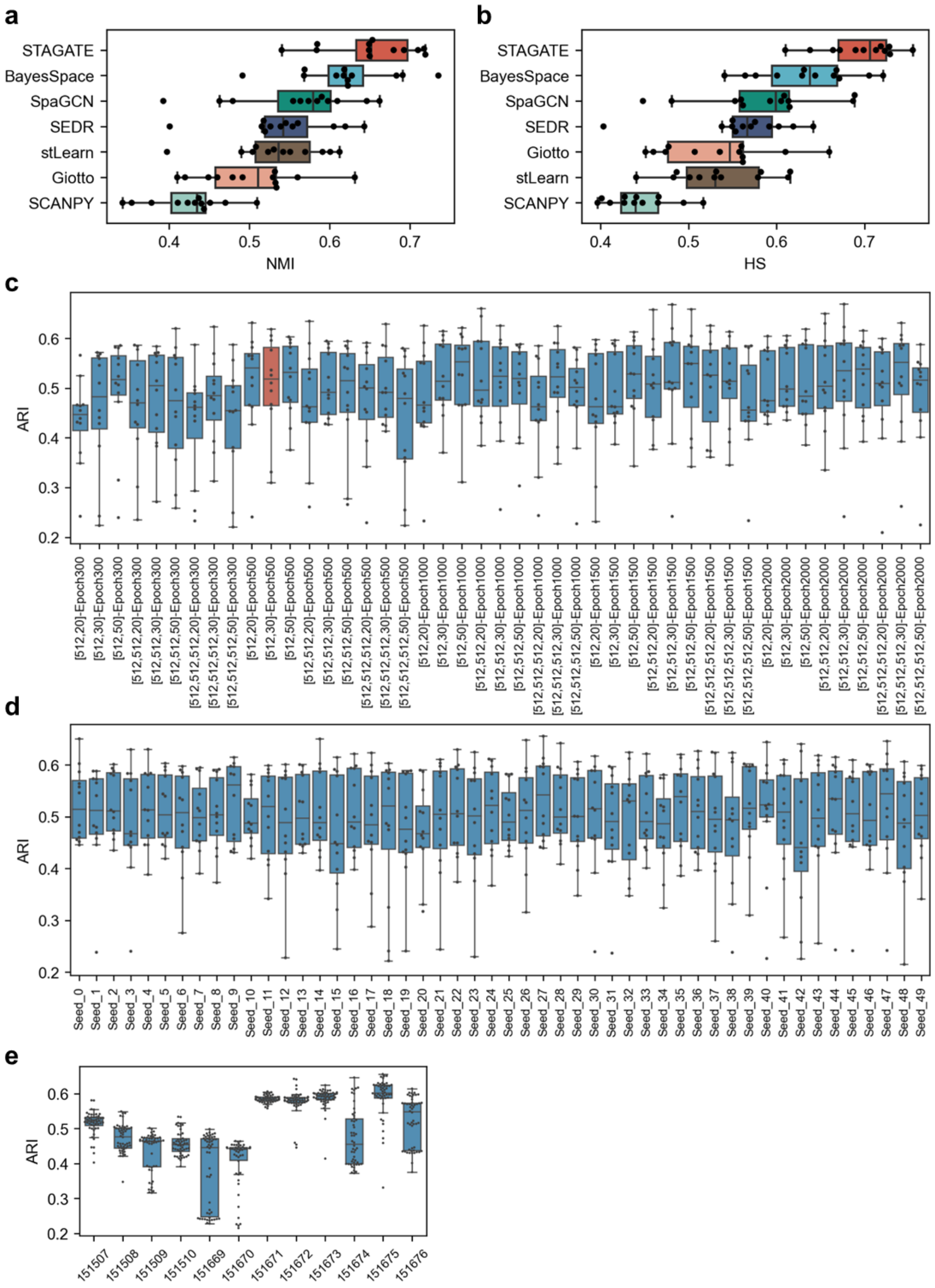

Fig. S2. Comparison of clustering accuracy on the DLPFC dataset. a, Boxplot of clustering accuracy in all the 12 sections of the DLPFC dataset in terms of normalized mutual information (NMI). **b,** Boxplot of clustering accuracy in all 12 sections of the DLPFC dataset in terms of homogeneity score (HS). **c,** The clustering accuracy of STAGATE with different hyperparameters in all 12 sections. The hyperparameters are selected by a grid search on three hyperparameters of the encoder layer number (2, 3 or 4), the latent dimension (20, 30 or 50) and the number of epochs (500, 1000, 1500, 2000). **d,** The clustering accuracy of STAGATE in all 12 sections under the default hyperparameters with different random seeds. **e**, The clustering accuracy for each section under the default hyperparameters with different random seeds (n=50 for each box). In all boxplots, the center line, box limits and whiskers denote the median, upper and lower quartiles and 1.5× interquartile range, respectively.

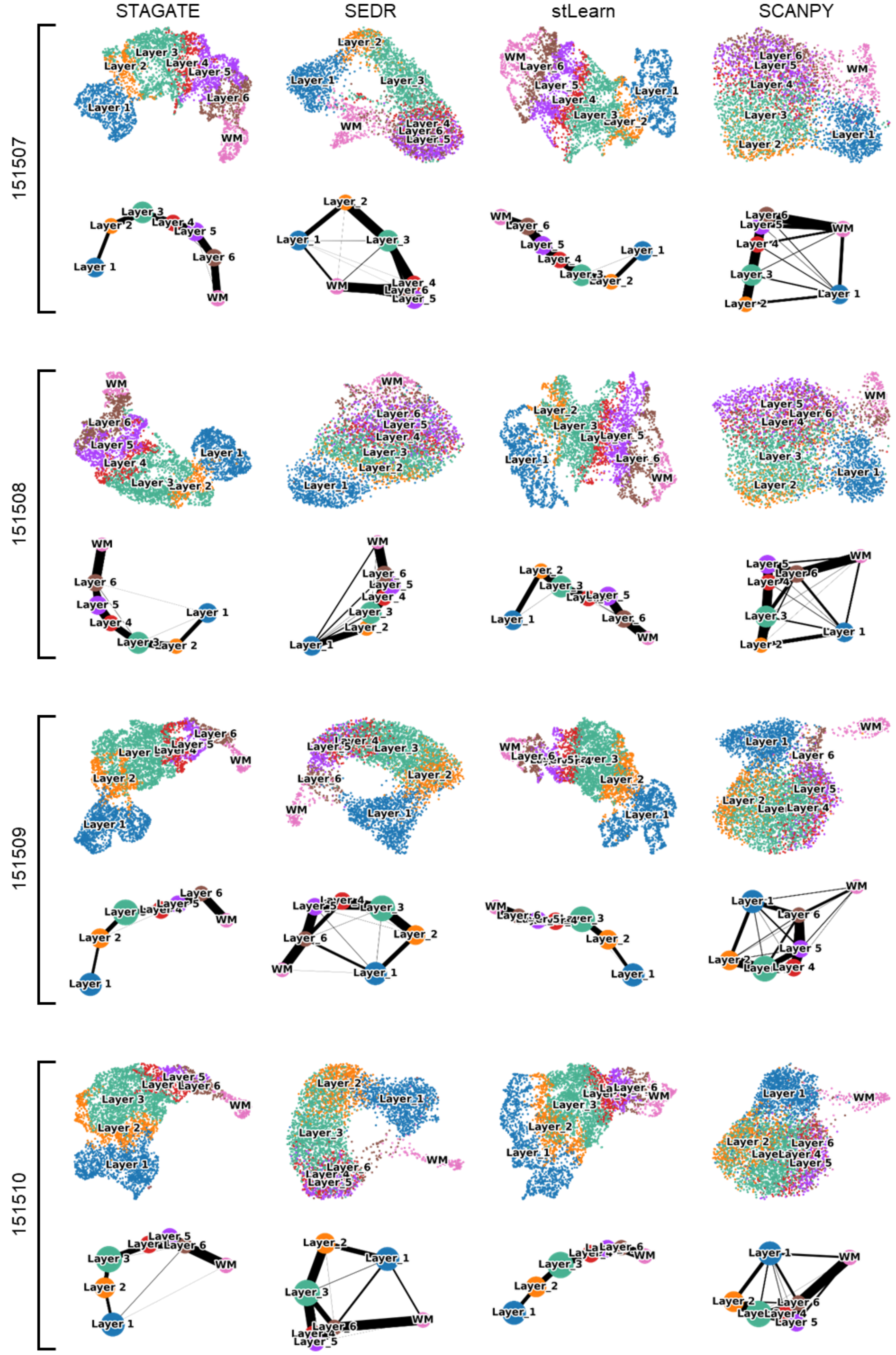

See next page

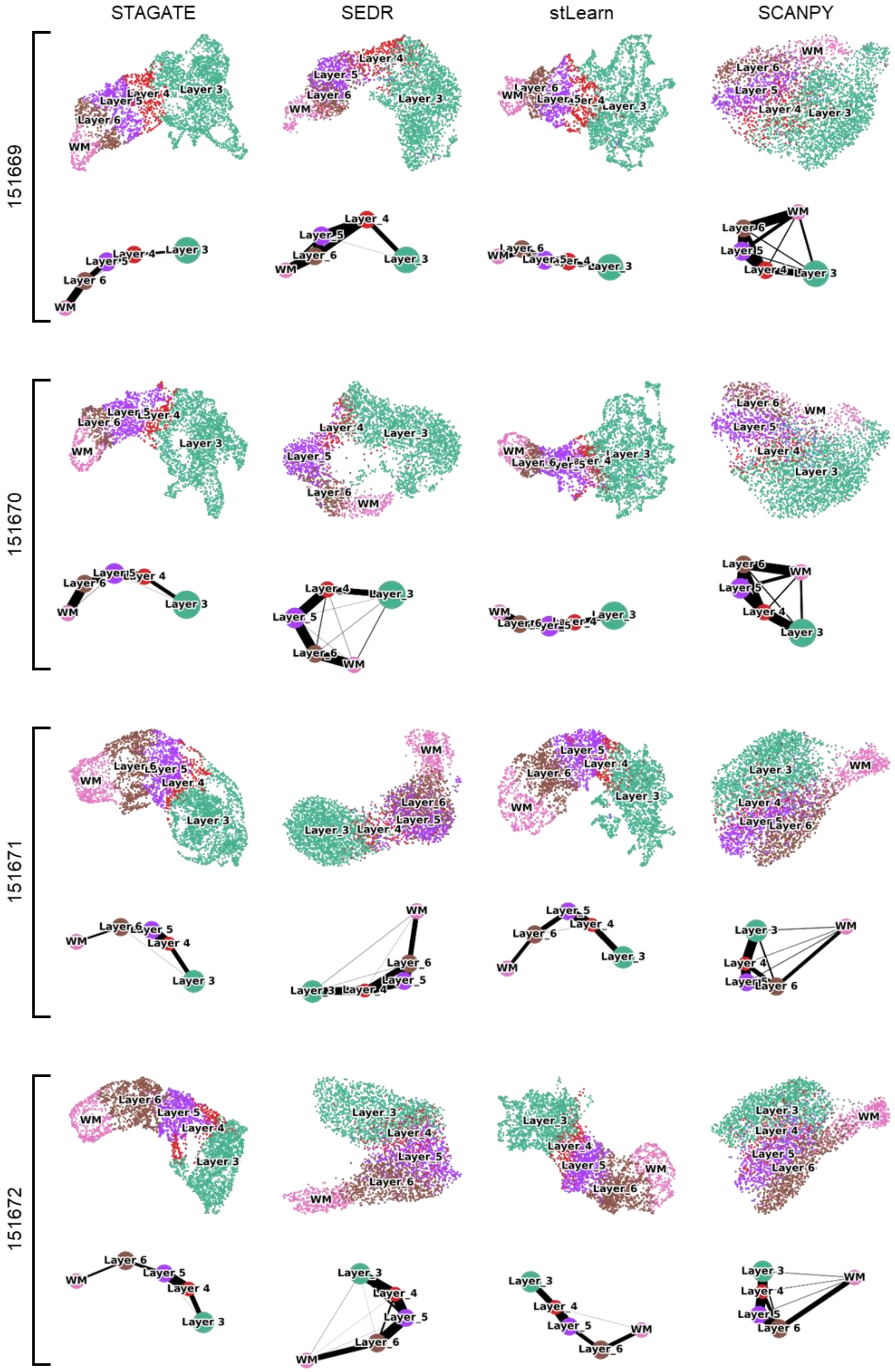

See next page

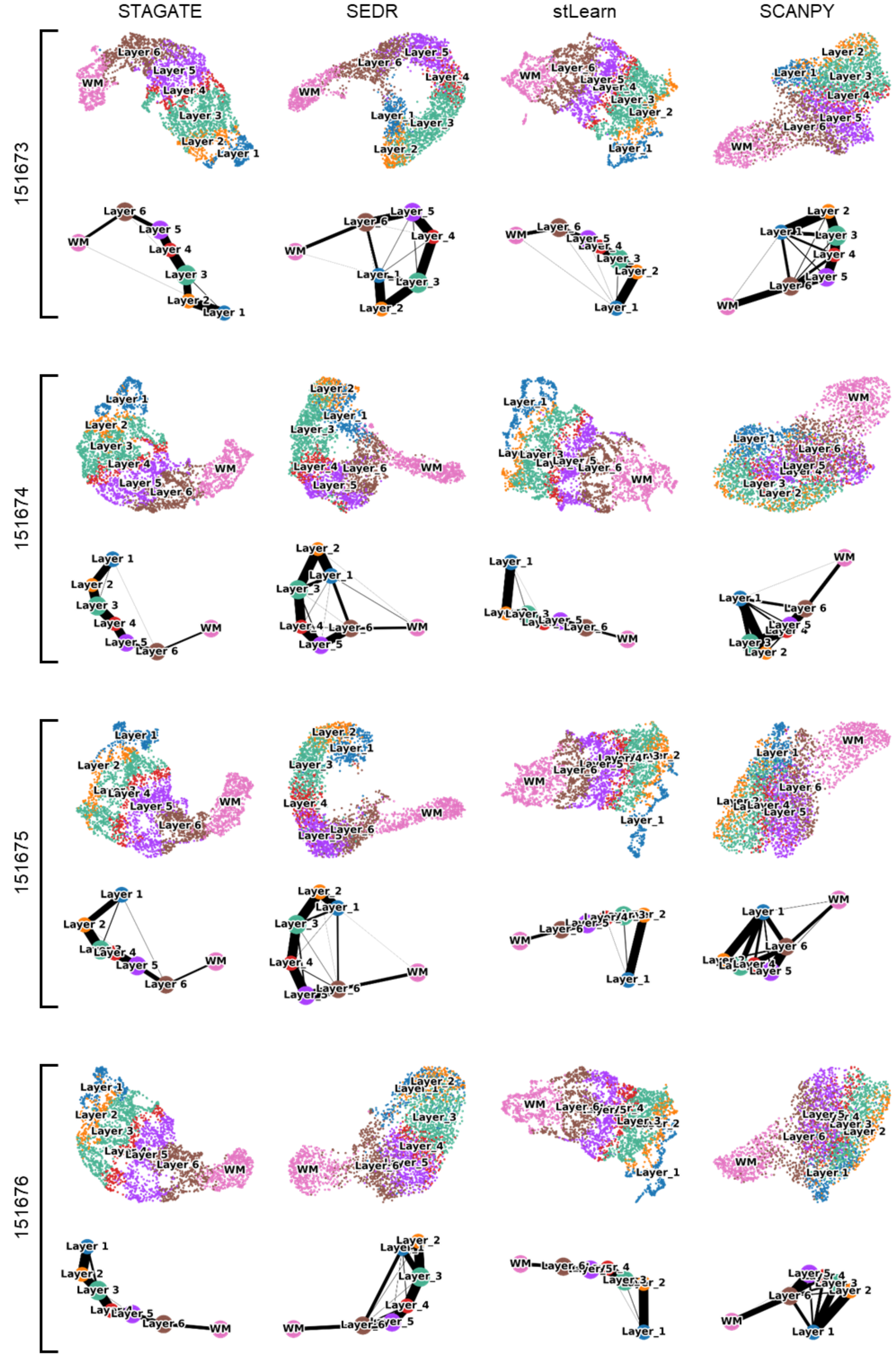

Fig. S3. UMAP visualization and PAGA graphs generated by STAGATE, SEDR, stLearn, and SCANPY embeddings respectively. Spots are colored by their manual annotations.

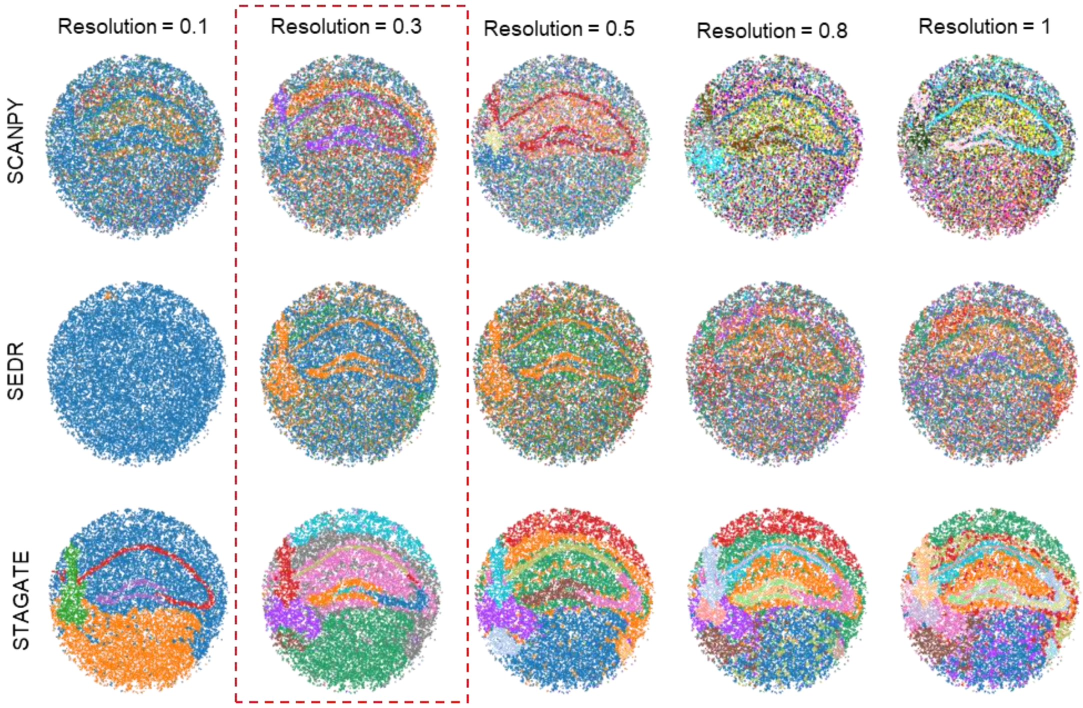

Fig. S4. Comparison of spatial domains by applying Louvain clustering with different resolutions (0.1, 0.3, 0.5, 0.8, and 1) to the SCANPY, SEDR, STAGATE embeddings respectively in the Slide-seqV2 hippocampus data.

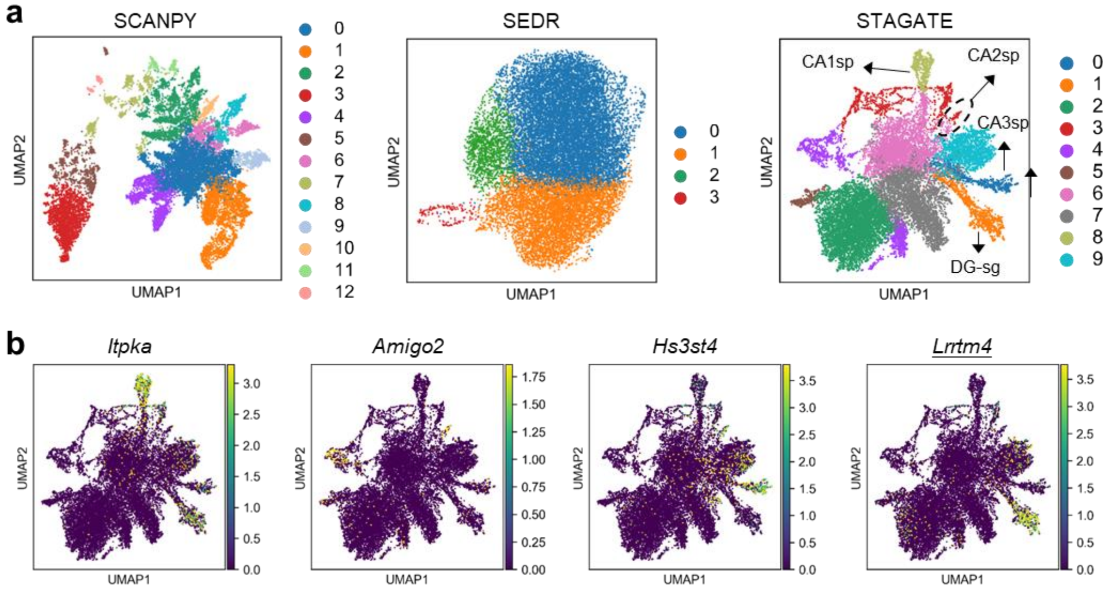

Fig. S5. Comparison of the UMAP visualization of SCANPY, SEDR, and STAGATE in the Slide-seqV2 hippocampus data. a, UMAP visualization generated by SCANPY, SEDR, and STAGATE embeddings respectively. Spots are colored by their spatial domains identified by the corresponding approach. **b**, UMAP visualization colored by expressions of ITPKA, AMIGO2, HS3ST4, and LRRTM4 respectively.

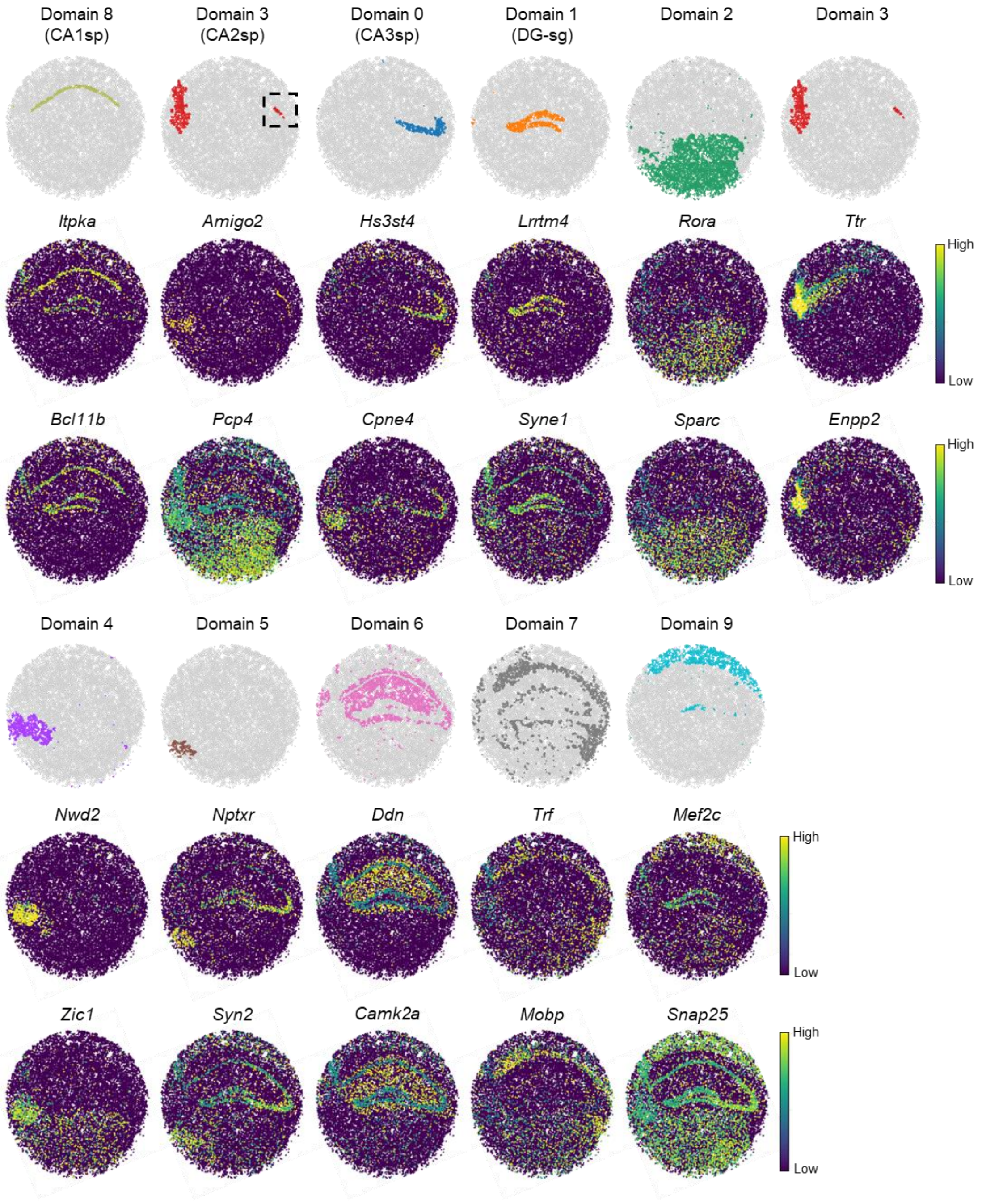

Fig. S6. Visualizations of spatial domains identified by applying Louvain clustering with resolution=0.3 to the STAGATE embedding and expressions of the corresponding marker genes in the Slide-seqV2 data of the mouse hippocampus tissue.

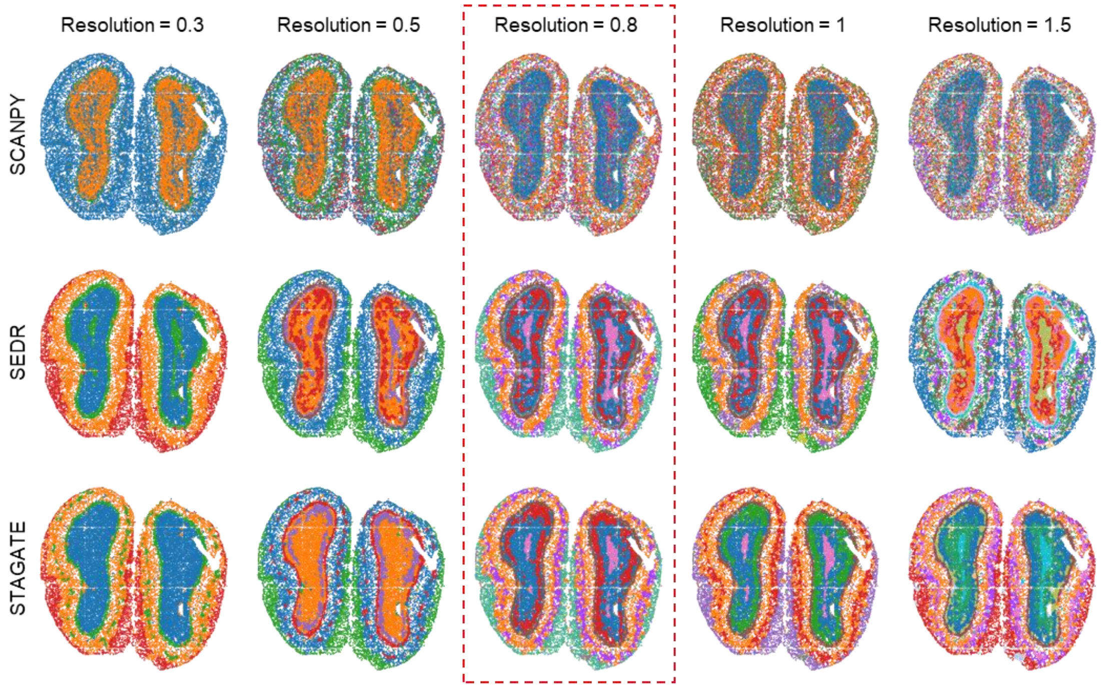

Fig. S7. Comparison of spatial domains by applying Louvain clustering with different resolutions (0.3, 0.5, 0.8, 1, and 1.5) to the SCANPY, SEDR, and STAGATE embeddings respectively in the Stereo-seq mouse olfactory bulb tissue section.

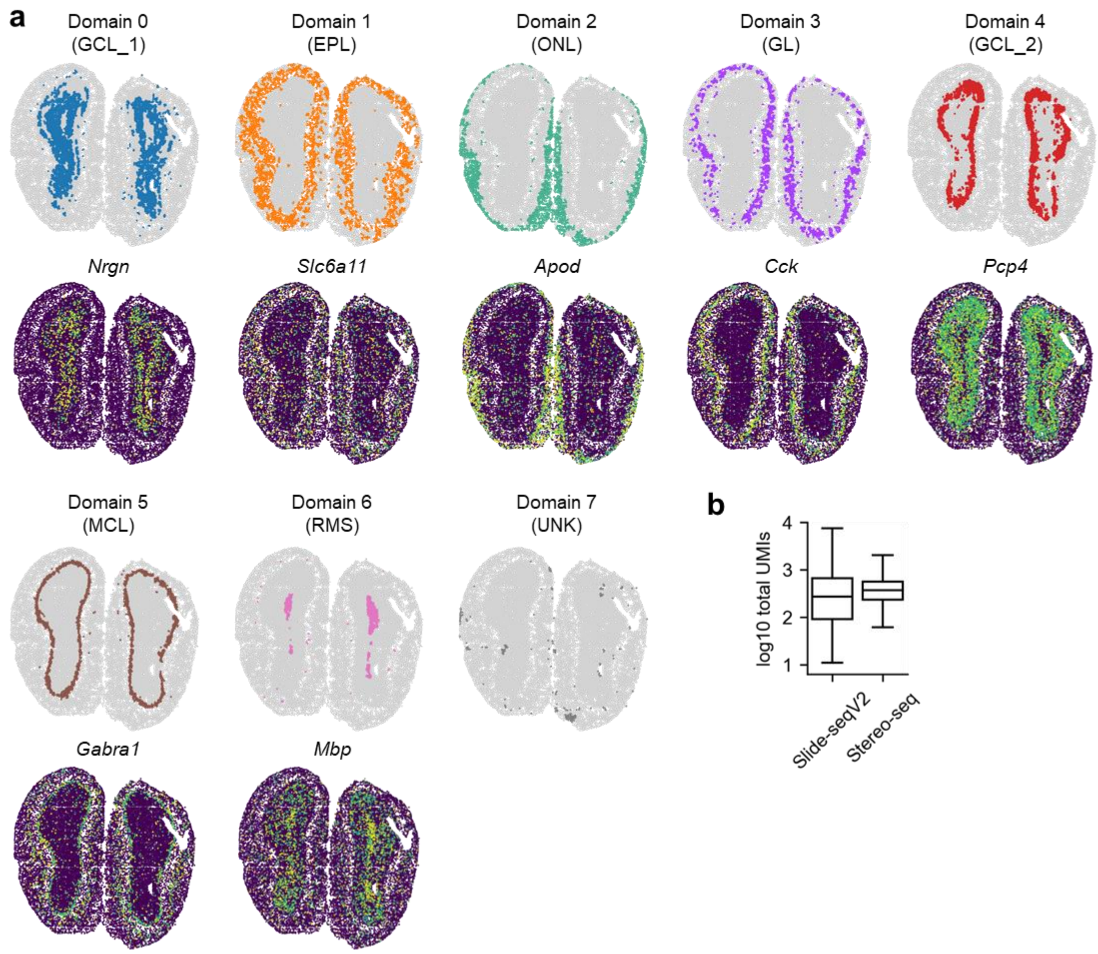

Fig. S8. Visualizations of spatial domains identified by applying Louvain clustering with resolution=0.8 to the STAGATE embedding and expressions of the corresponding marker genes in the Stereo-seq mouse olfactory bulb tissue section. b, Number of total UMIs per spot in the mouse olfactory bulb tissue sections generated by Slide-seqV2 (n=20,139 spots) and Stereo-seq (n=19,109 spots) respectively. In the boxplot, the center line, box limits and whiskers denote the median, upper and lower quartiles and 1.5× interquartile range, respectively.

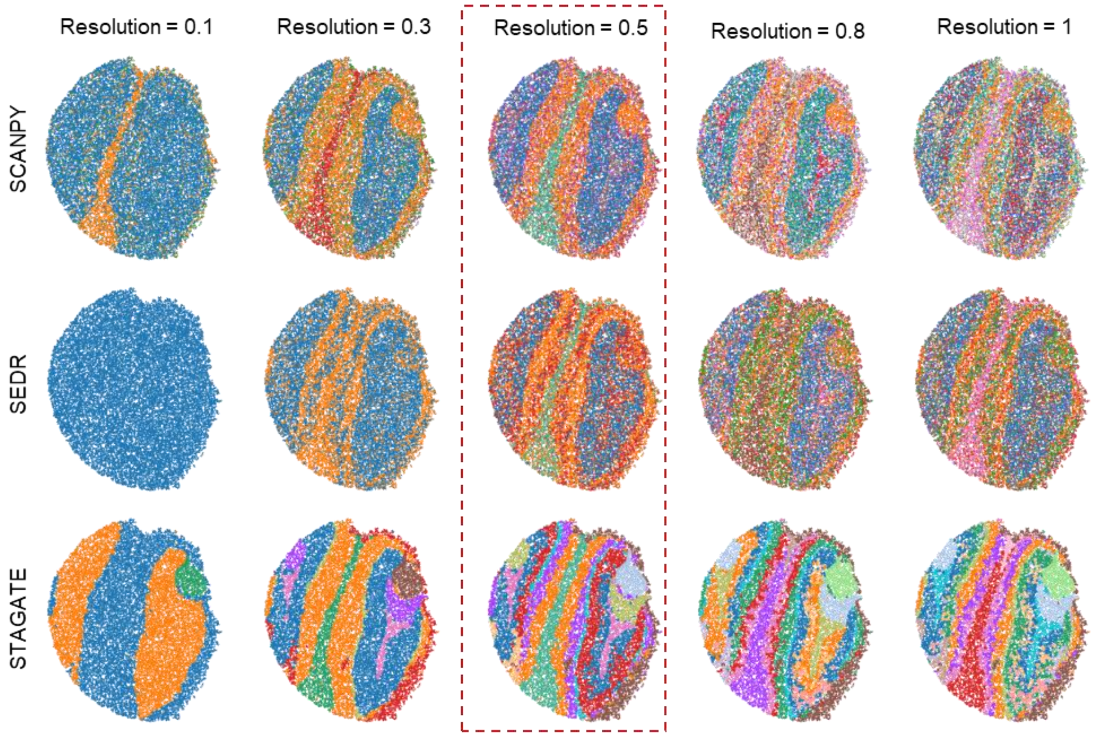

Fig. S9. Comparison of spatial domains identified by applying Louvain clustering with different resolutions (0.1, 0.3, 0.5, 0.8, and 1) to the SCANPY, SEDR, and STAGATE embeddings respectively on the Slide-seqV2 data of mouse olfactory bulb tissue section.

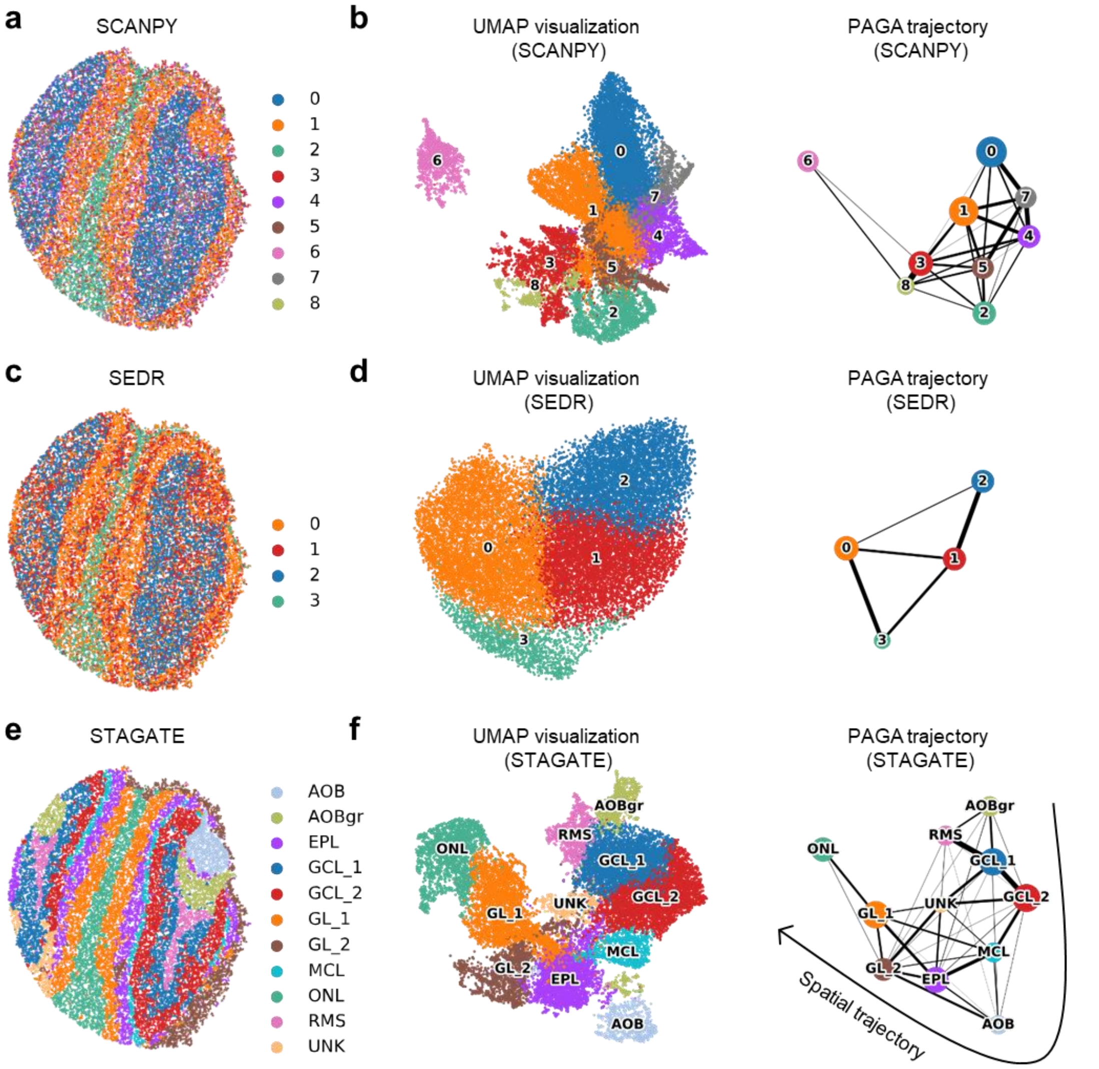

Fig. S10. Spatial trajectory inference in the Slide-seqV2 data of mouse olfactory bulb tissues. **a**, Spatial domains identified by SCANPY embedding using Louvain clustering with resolution=0.5. **b**, UMAP visualization and PAGA graph generated based on the SCANPY embedding. **c**, Spatial domains identified by SEDR embedding using Louvain clustering with resolution=0.5. **d**, UMAP visualization and PAGA graph generated based on the STAGATE embedding. **e**, Spatial domains identified by SEDR embedding using Louvain clustering with resolution=0.5. **f**, UMAP visualization and PAGA graph generated based on the STAGATE embedding.

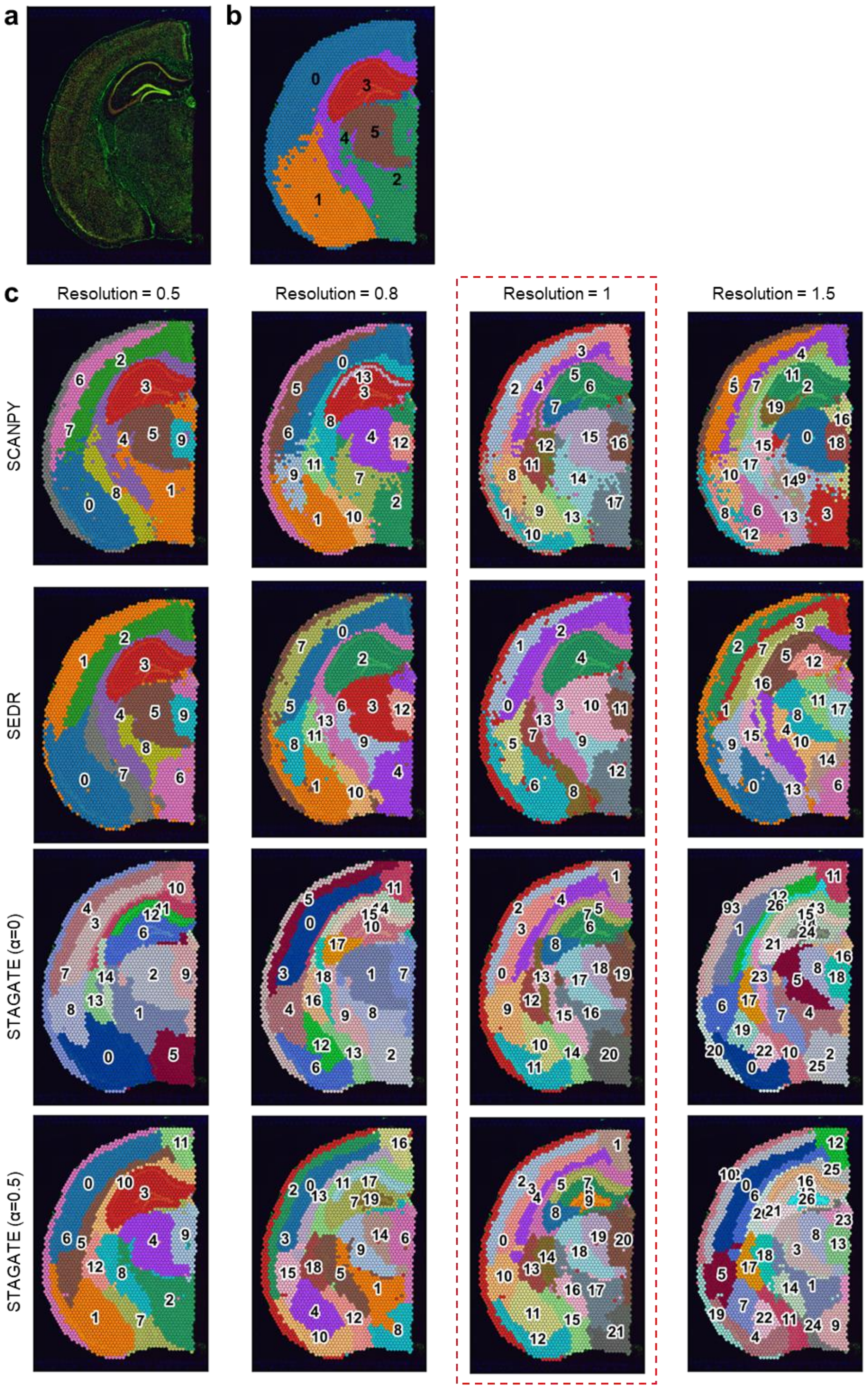

Fig. S11. Comparison of spatial domains identified by different methods in the adult mouse brain section profiled by 10x Visium. a, Immunofluorescent imaging of the tissue section stained with DAPI and Anti-NeuN. **b**, Spatial domains identified by applying Louvain clustering with resolution 0.2 to the spatial gene expressions directly. **c**, Spatial domains identified by applying Louvain clustering with different resolutions (0.5, 0.8, 1, 1.5) to the SCANPY, SEDR, and STAGATE (α=0 and α=0.5) embeddings in the adult mouse brain section profiled by 10x Visium.

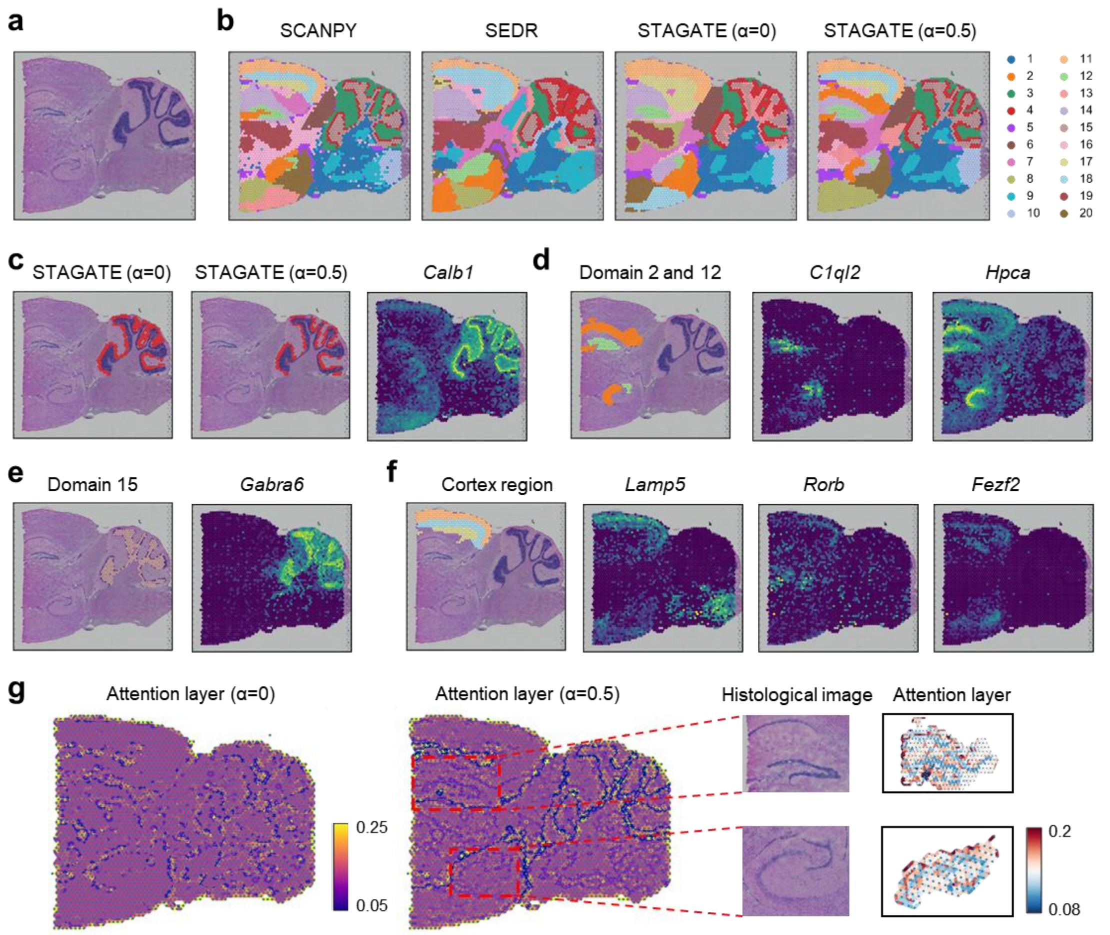

Fig. S12. STAGATE reveals spatial domains in adult mouse posterior brain section profiled by 10x Visium. a, Histological image of the tissue section. **b,** Spatial domains generated by mclust clustering on the low-dimensional embeddings of SCANPY, SEDR, STAGATE, and STAGATE with the cell type-aware module. The α represents the weight of cell type-aware SNN (see **Figure 1**). **c,** Comparison of the thin spatial domain around the coronal structure identified by STAGATE without or with the cell type-aware module. **d-f,** Visualization of spatial domains identified by STAGATE using the cell type-aware module and the corresponding marker genes. **g,** Visualizations of the attention layer of STAGATE without or with the cell type-aware module. The nodes of the attention layer are arranged according to the spatial position of spots. The edges of the attention layer are colored by corresponding weights.

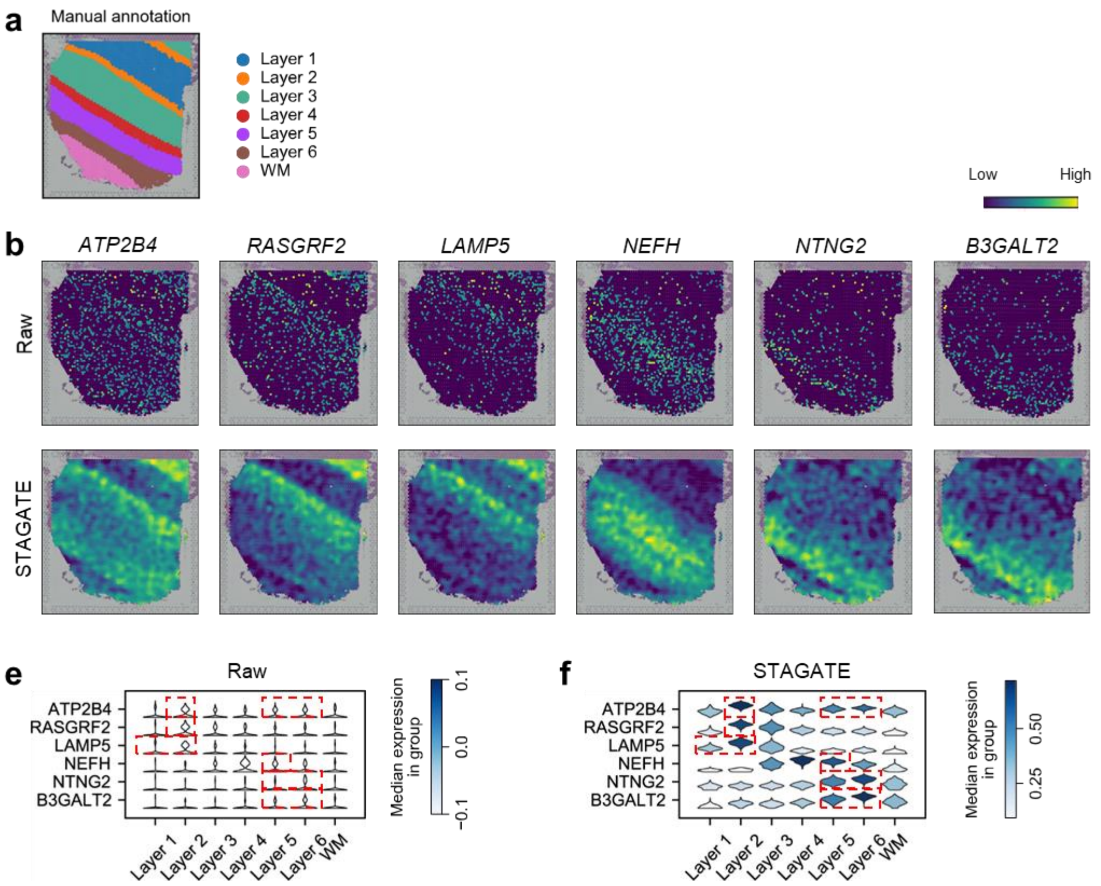

Fig. S13. Comparison of the spatial expression patterns before and after STAGATE denoising. a, Ground-truth segmentation of cortical layers and white matter (WM) in the DLPFC section 151507. **b**, Expression visualization of six layer-marker genes in the DLPFC section 151507. **c** and **d**, Violin plots of the raw expressions (**c**) and the STAGATE denoised expressions (**d**) of layer-marker genes in the DLPFC section 151507 respectively. The cortical layer corresponding to the layer-marker genes is marked with red boxes.

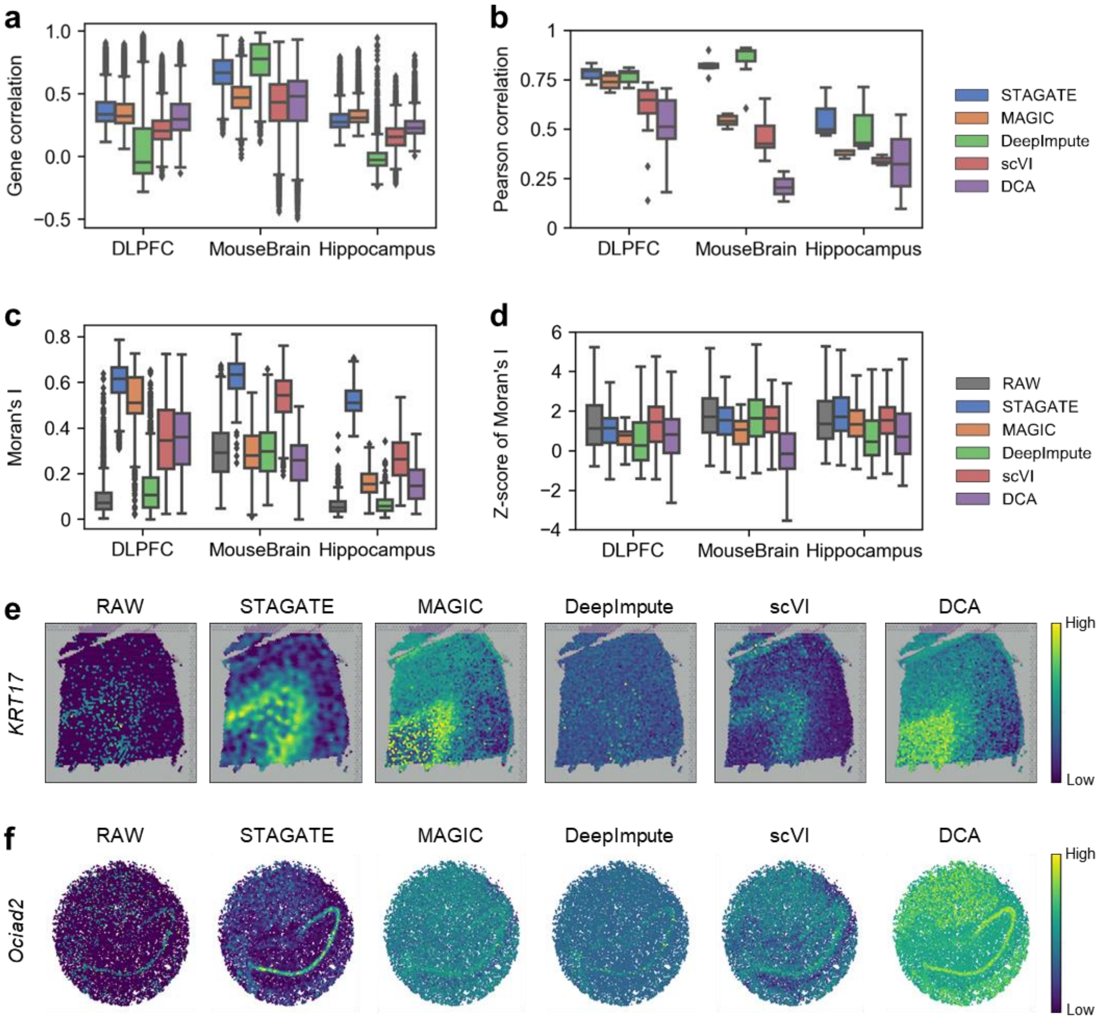

Fig. S14. Evaluation of imputation efficiency by downsampling the raw data. a and **b**, Gene-wise Spearman correlation (**a**) and Pearson correlation (**b**) values of all downsampling pairs in each section of the DLPFC dataset (10x Visium platform, 12 sections), the MouseBrain dataset (10x Visium platform, seven sections), and the Hippocampus dataset (Slide-seqV2 platform, three sections). **c** and **d**, Moran's I values (**c**) and the normalized Moran's I values (**d**) of the first 100 spatially variable genes identified by SPARK-X in the observed/imputed matrix (n=100 genes for each box). The outliers in the boxplot of (**d**) are ignored. **e**, Visualizations of KRT17 in the raw and imputed data in the DLPFC section 151676 respectively. **f**, Visualizations of OCIAD2 in the raw and imputed data in Slide-seqV2 Hippocampus data respectively. In all boxplots, the center line, box limits and whiskers denote the median, upper and lower quartiles and 1.5× interquartile range, respectively.

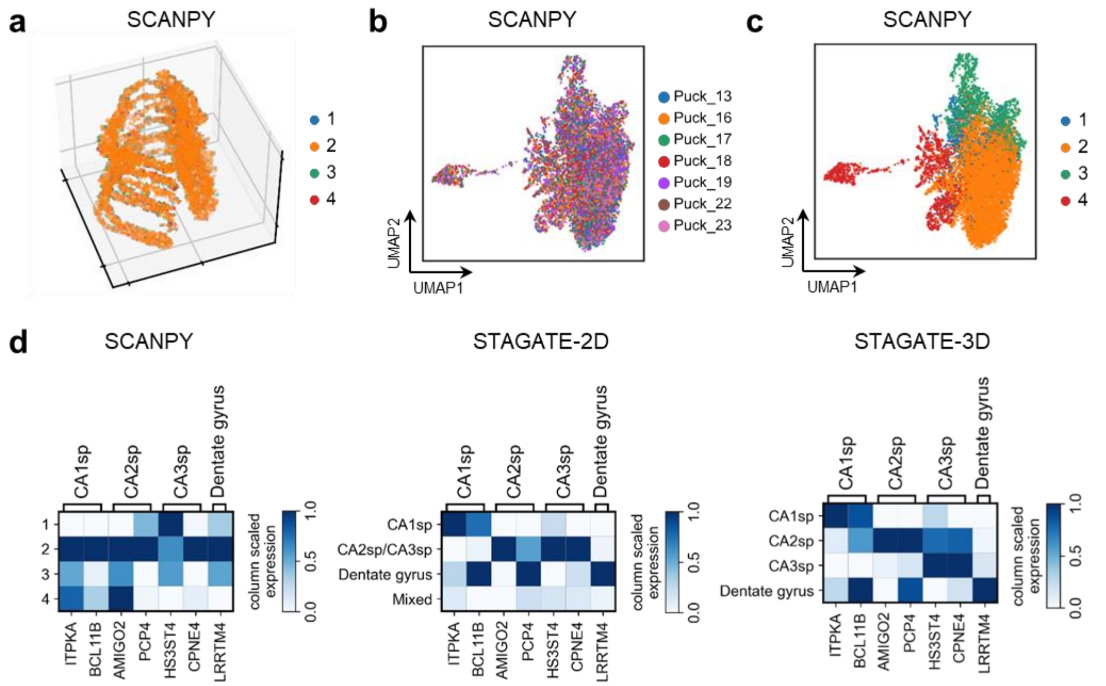

Fig. S15. Spatial domains identified by applying mclust clustering to the SCANPY embedding in the 3D hippocampus model. a, Cluster assignments generated using mclust clustering on the SCANPY embedding. **b**, UMAP visualization generated by SCANPY embedding colored by section IDs. **c**, UMAP visualization generated by SCANPY embedding colored by clustering labels. **d,** Mean expressions of marker genes across spatial domains identified by SCANPY, STAGATE-2D, and STAGATE-3D. The mean expressions were scaled according to columns.

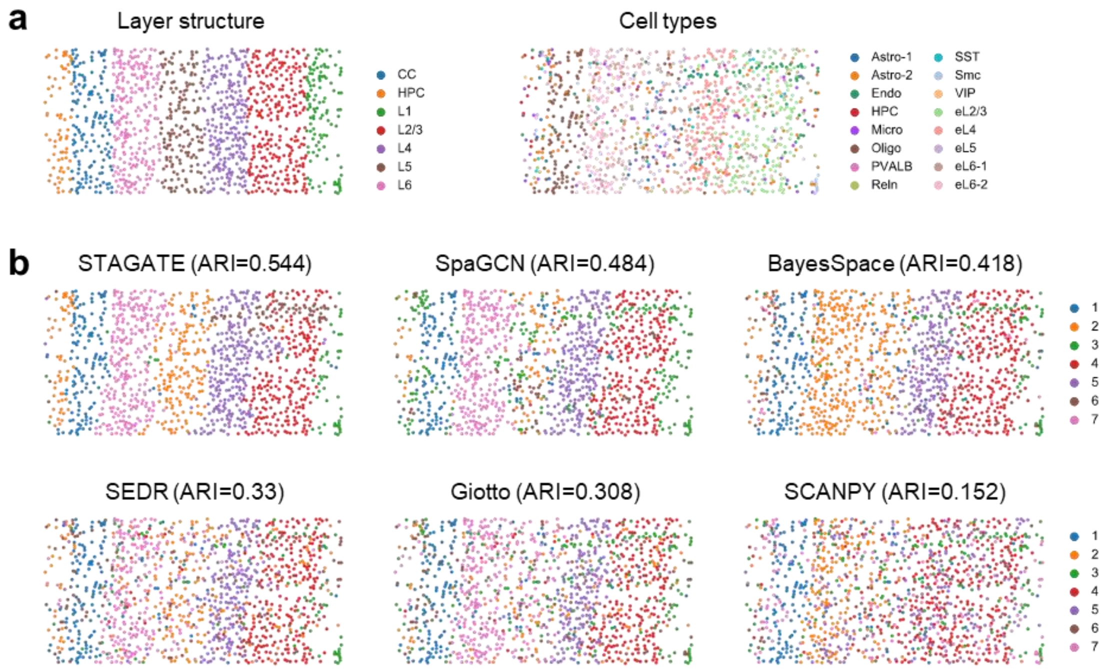

Fig. S16. Spatial domains detected in the mouse visual cortex STARmap data. a, Layer structure and cell type distribution of the tissue section from the original study. **b,** Spatial domains identified by STAGATE, SpaGCN, BayesSpace, SEDR, Giotto and SCANPY respectively.

a

Section	Platform	#Spots	#Edges	#Time	Memory
DLPFC 151676	10X Visium	3,460	10,026	29.3s	0.71 GB
MouseBrain	10X Visium	2,903	8,491	63.6s	0.65 GB
Stereo-seq	Stereo-seq	19,109	72,159	748s	2.23 GB
Puck_190921_21	Slide-seqV2	19,285	113,219	814.7s	2.23GB
Puck_200127_15	Slide-seqV2	20,139	114,144	848.8s	2.11 GB
Puck_191204_01	Slide-seqV2	33,721	142,595	1536.8s	4.25 GB
Puck_200115_08	Slide-seqV2	52,869	189,074	2364s	6.39 GB

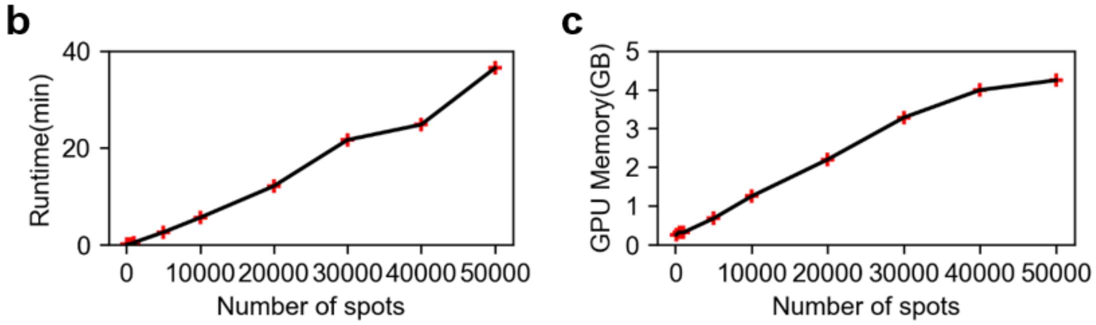

Fig. S17. Running time and GPU memory usage. a and **b**, Running time and GPU memory usage on the simulated datasets referring to the DLPFC dataset with different numbers of spots based on GeForce GTX 1080 GPU. The spots were arranged in the form of 10X Visium sections. **c**, Running time and GPU memory usage in the real datasets. The network architecture of all experiments is set as 3000-512-30.

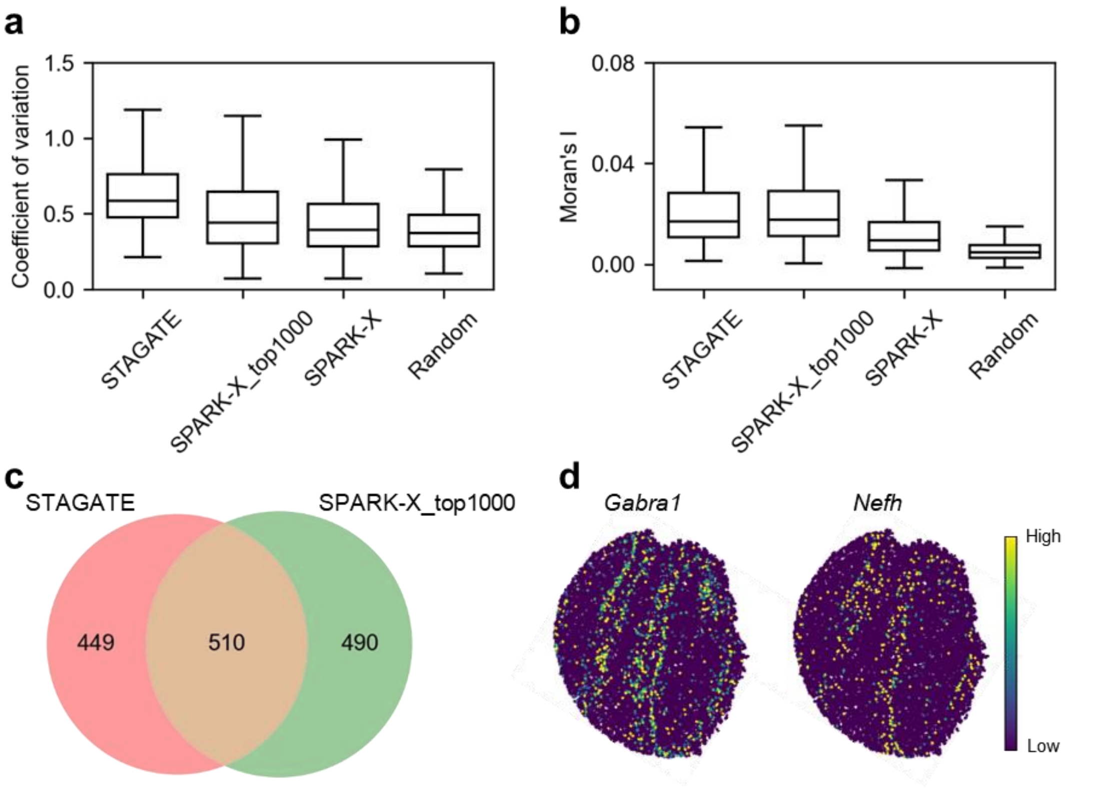

Fig. S18. Comparison of the spatial domain-specific expression patterns of STAGATE and SPARK-X. a, Boxplot of the coefficient of variation (CV) of genes across spatial domains in the Slide-seqV2 data of the mouse olfactory bulb tissue. **b**, Boxplot of Moran's I values for spatially variable genes (SVGs) detected by STAGATE and SPARK-X respectively. The number of detected genes of STAGATE, SPARK-X_top1000, SPARK-X and Random are 959, 1,000, 2,479 and 1,000 respectively. **c**, Venn diagram of SVGs identified by STAGATE and the first 1000 SVGs detected by SPARK-X. **d**, Visualizations of two specifically expressed genes of the MCL domain. In all boxplots, the center line, box limits and whiskers denote the median, upper and lower quartiles and 1.5× interquartile range, respectively.

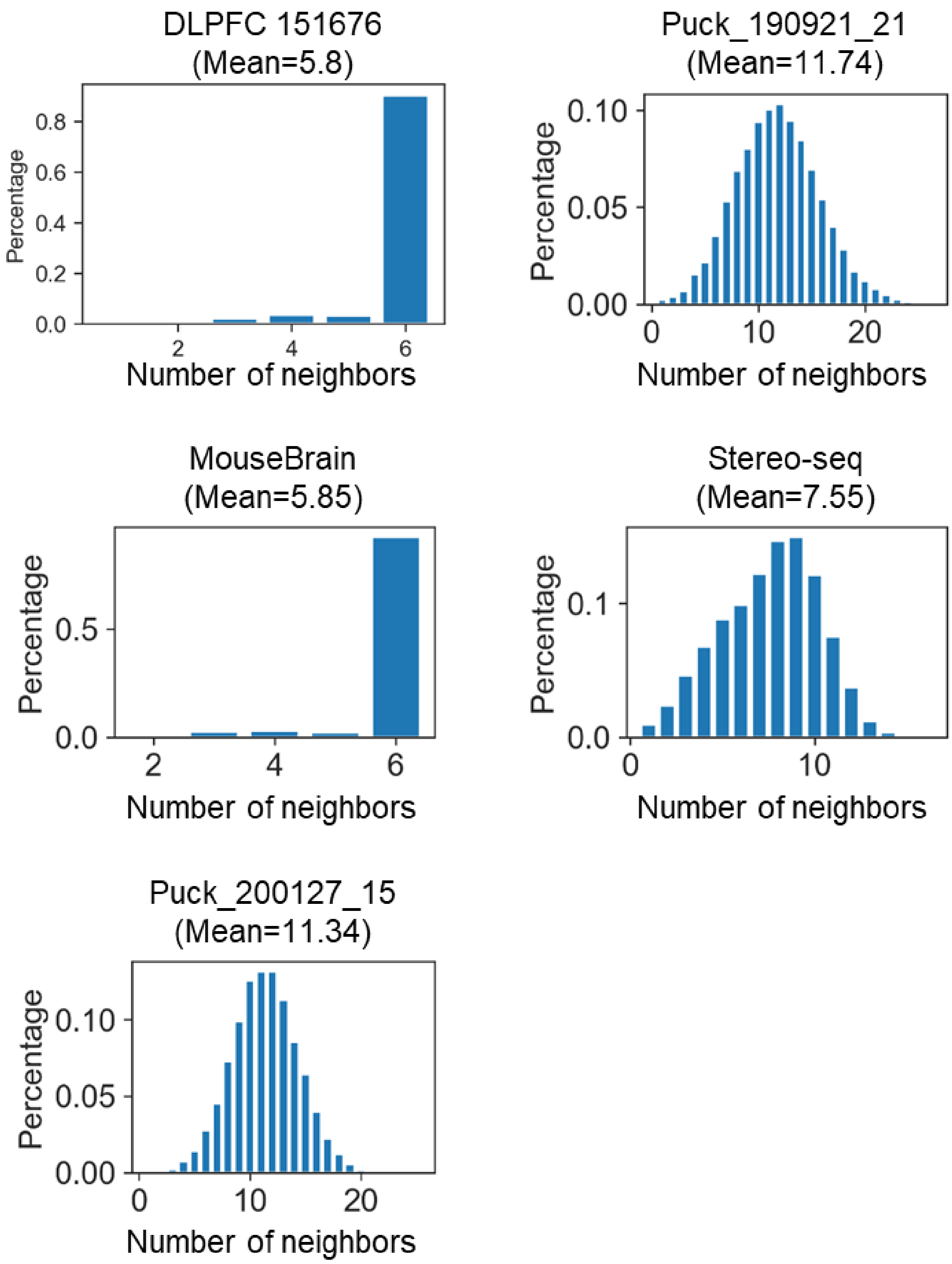

Fig. S19. Statistics of the number of neighbors per spot in SNNs.

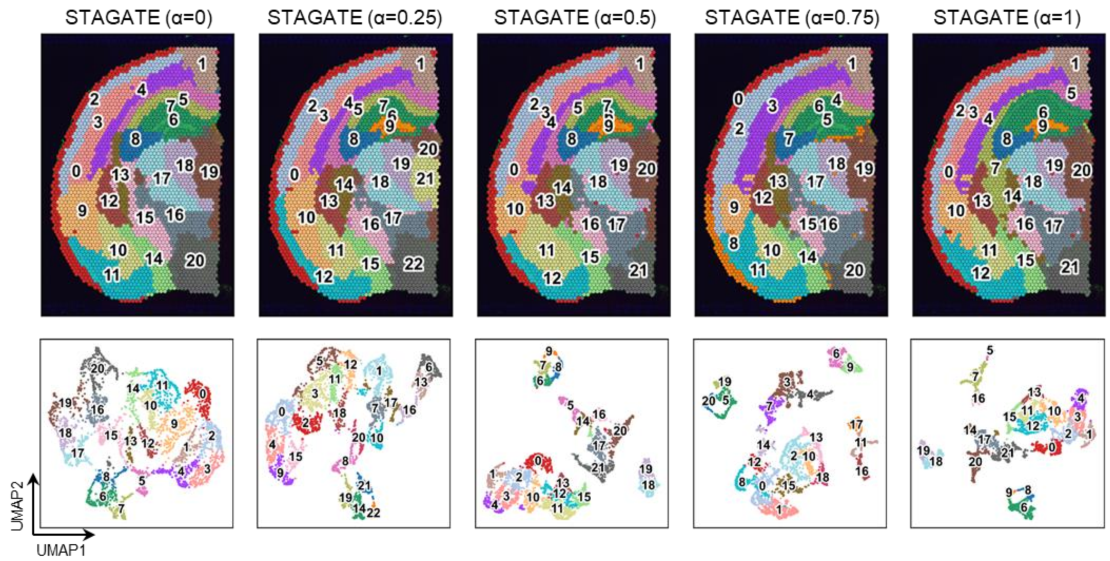

Fig. S20. Spatial domains and UMAP visualization generated by STAGATE under different α values.

Table S1. Comparison between current bioinformatics methods for spatial domain identification of ST data.

Method	Spatial information integration	Histology image usage	Scalability (~100k spots)	Spatial trajectory inference	Data denosing	Super-resolution	Adaptively learn spatial similarity	Support 3D ST model
SCANPY			√					
Giotto	√							
BayesSpace	√					√		
stLearn	√	√	√	√	√			
SEDR	√		√	√				
SpaGCN	√	√	√					
STAGATE	√		√	√	√		√	√

Table S2. Description of all ST datasets used in this study.

Platform	Tissue	Section	#Spots	Related figures	Reference
10X Visium	human dorsolateral prefrontal cortex (DLPFC)	151507, 151508, 151509, 151510, 151669, 151670, 151671, 151672, 151673, 151674, 151675, 151676.	4226, 4384, 4789, 4634, 3661, 3498, 4110, 4015, 3639, 3673, 3592, 3460.	Fig. 2a-d, Fig. 6, Fig. S1, S2, S11, S12	[17]
	MouseBrain	Adult Mouse Brain Section 1 (Coronal)	2903	Fig. 5, Fig. S10, Fig. S12a-d, Fig. S18	10x Visium demo
		Adult Mouse Brain Section 2 (Coronal)	2807	Fig. S12a-d	
		Mouse Brain Section (Coronal)	2702	Fig. 3f, Fig. S12a-d	
		Mouse Brain Section 1 (Sagittal-Anterior)	2695	Fig. S12a-d	
		Mouse Brain Section 1 (Sagittal-Posterior)	3355	Fig. S12a-d	
		Mouse Brain Section 2 (Sagittal-Anterior)	2825	Fig. S12a-d	
		Mouse Brain Section 2 (Sagittal-Posterior)	3289	Fig. S12a-d	
Slide-seqV2	Hippocampus	Puck_190921_21	19285	Fig. 3a, c Fig. S3, S4, S5 Fig. S12a-d, f	[8]
		Puck_191204_01	33721	Fig. S12a-d	
		Puck_200115_08	52869	Fig. S12a-d	
	Mouse olfactory bulb	Puck_200127_15	20139	Fig. 4d, e Fig. S6, S7, S12	
Slide-seq	Hippocampus	Puck_180531_23	18509	Fig. 3f	[7]
Stereo-seq	Mouse olfactory bulb	X	19109	Fig. 4a, b Fig. S6, S7	[9]
STARMAP	Mouse visual cortex	X	1207	Fig. S16	[18]

Table S3. Description of Slide-seq data used to reconstruct the 3D hippocampus model.

Platform	Tissue	Section	#Total spots	#Used spots	Reference
Slide-seq	Hippocampus	Puck_180531_13	32614	1692	[7]
		Puck_180531_16	20517	1476	
		Puck_180531_17	19140	1138	
		Puck_180531_18	28945	1363	
		Puck_180531_19	31139	1788	
		Puck_180531_22	29707	1835	
		Puck_180531_23	23841	1616	

Table S4. Sample IDs and URLs of data downloaded from the Allen Brain Atlas in this study.

Related figures	ABA ID	ABA URL
Fig. 3b	100960088	atlas.brain-map.org/atlas?atlas=1&plate=100960088
Fig. 4c	100960460	atlas.brain-map.org/atlas?atlas=1&plate=100960460
Fig. 6b (ATP2B4)	78721333	human.brain-map.org/ish/specimen/show/78721333?gene=490
Fig. 6b (RASGRF2)	78720797	human.brain-map.org/ish/specimen/show/78720797?gene=5892
Fig. 6b (LAMP5)	80324388	human.brain-map.org/ish/specimen/show/80324388?gene=23892
Fig. 6b (NEFH)	80393601	human.brain-map.org/ish/specimen/show/80393601?gene=4718
Fig. 6b (NTNG2)	78720797	human.brain-map.org/ish/specimen/show/78720797?gene=58827
Fig. 6b (B3GALT2)	78721333	human.brain-map.org/ish/specimen/show/78721333?gene=8637

Reference

1. Wolf, F.A., Angerer, P. & Theis, F.J. SCANPY: large-scale single-cell gene expression data analysis. *Genome Biol.* **19**, 1-5 (2018).
2. Dries, R. et al. Giotto: a toolbox for integrative analysis and visualization of spatial expression data. *Genome Biol.* **22**, 1-31 (2021).
3. Zhao, E. et al. Spatial transcriptomics at subspot resolution with BayesSpace. *Nat. Biotechnol.* 1-10 (2021).
4. Pham, D. et al. stLearn: integrating spatial location, tissue morphology and gene expression to find cell types, cell-cell interactions and spatial trajectories within undissociated tissues. *bioRxiv* (2020). doi: https://doi.org/10.1101/2020.05.31.125658.
5. Hu, J. et al. SpaGCN: Integrating gene expression, spatial location and histology to identify spatial domains and spatially variable genes by graph convolutional network. *Nat. Methods* **18**, 1342-1351 (2021).
6. Chen, J. et al. Unsupervised Spatially Embedded Deep Representation of Spatial Transcriptomics. *bioRxiv* (2021). doi: https://doi.org/10.1101/2021.06.15.448542.
7. Rodriques, S.G. et al. Slide-seq: A scalable technology for measuring genome-wide expression at high spatial resolution. *Science* **363**, 1463-1467 (2019).
8. Stickels, R.R. et al. Highly sensitive spatial transcriptomics at near-cellular resolution with Slide-seqV2. *Nat. Biotechnol.* **39**, 313-319 (2021).
9. Chen, A. et al. Large field of view-spatially resolved transcriptomics at nanoscale resolution. *bioRxiv* (2021). doi: https://doi.org/10.1101/2021.01.17.427004.
10. Van Dijk, D. et al. Recovering gene interactions from single-cell data using data diffusion. *Cell* **174**, 716-729. e727 (2018).
11. Arisdakessian, C., Poirion, O., Yunits, B., Zhu, X. & Garmire, L.X. DeepImpute: an accurate, fast, and scalable deep neural network method to impute single-cell RNA-seq data. *Genome Biol.* **20**, 1-14 (2019).
12. Lopez, R., Regier, J., Cole, M.B., Jordan, M.I. & Yosef, N. Deep generative modeling for single-cell transcriptomics. *Nat. Methods* **15**, 1053-1058 (2018).
13. Eraslan, G., Simon, L.M., Mircea, M., Mueller, N.S. & Theis, F.J. Single-cell RNA-seq denoising using a deep count autoencoder. *Nat. Commun.* **10**, 1-14 (2019).
14. Li, H., Calder, C.A. & Cressie, N. Beyond Moran's I: testing for spatial dependence based on the spatial autoregressive model. *Geogr. Anal.* **39**, 357-375 (2007).
15. Nitzan, M., Karaiskos, N., Friedman, N. & Rajewsky, N. Gene expression cartography. *Nature* **576**, 132-137 (2019).
16. Stuart, T. et al. Comprehensive integration of single-cell data. *Cell* **177**, 1888-1902. e1821 (2019).
17. Maynard, K.R. et al. Transcriptome-scale spatial gene expression in the human dorsolateral prefrontal cortex. *Nat. Neurosci.* **24**, 425-436 (2021).
18. Wang, X. et al. Three-dimensional intact-tissue sequencing of single-cell transcriptional states. *Science* **361** (2018).
