# Supplementary material for: Deciphering spatial domains from spatially resolved transcriptomics with an adaptive graph attention auto-encoder
Source: Nat Commun. 2022 Apr 1;13:1739. doi: 10.1038/s41467-022-29439-6 (PMC8976049; doi:10.1038/s41467-022-29439-6)
Supplement: Supplementary file 2 — Reporting Summary [file 41467_2022_29439_MOESM2_ESM.pdf]

nature portfolio

Corresponding author(s): Shihua Zhang

Last updated by author(s): Mar 5, 2022

Reporting Summary

Nature Portfolio wishes to improve the reproducibility of the work that we publish. This form provides structure for consistency and transparency in reporting. For further information on Nature Portfolio policies, see our Editorial Policies and the Editorial Policy Checklist.

Statistics

For all statistical analyses, confirm that the following items are present in the figure legend, table legend, main text, or Methods section.

n/a	Confirmed	
☐	☒	The exact sample size (n) for each experimental group/condition, given as a discrete number and unit of measurement
☒	☐	A statement on whether measurements were taken from distinct samples or whether the same sample was measured repeatedly
☐	☒	The statistical test(s) used AND whether they are one- or two-sided<br>*Only common tests should be described solely by name; describe more complex techniques in the Methods section.*
☒	☐	A description of all covariates tested
☒	☐	A description of any assumptions or corrections, such as tests of normality and adjustment for multiple comparisons
☐	☒	A full description of the statistical parameters including central tendency (e.g. means) or other basic estimates (e.g. regression coefficient) AND variation (e.g. standard deviation) or associated estimates of uncertainty (e.g. confidence intervals)
☐	☒	For null hypothesis testing, the test statistic (e.g. F, t, r) with confidence intervals, effect sizes, degrees of freedom and P value noted<br>*Give P values as exact values whenever suitable.*
☒	☐	For Bayesian analysis, information on the choice of priors and Markov chain Monte Carlo settings
☒	☐	For hierarchical and complex designs, identification of the appropriate level for tests and full reporting of outcomes
☐	☒	Estimates of effect sizes (e.g. Cohen's d, Pearson's r), indicating how they were calculated

Our web collection on statistics for biologists contains articles on many of the points above.

Software and code

Policy information about availability of computer code

Data collection	All data analyzed within this manuscript are publicly available. No additional software was used for the data collection process.
Data analysis	We performed all the data analysis using our developed STAGATE software (Version: 1.0.0) in this paper, which is available at [https://github.com/zhanglabtools/STAGATE]. STAGATE python package is built upon python 3.7.3. STAGATE have many dependencies, including scanpy v1.6.1, tensorflow v1.15.0, numpy v1.18.2, pandas v1.1.4, scipy v1.4.1, matplotlib v3.3.3.

For manuscripts utilizing custom algorithms or software that are central to the research but not yet described in published literature, software must be made available to editors and reviewers. We strongly encourage code deposition in a community repository (e.g. GitHub). See the Nature Portfolio guidelines for submitting code & software for further information.

Data

Policy information about availability of data

All manuscripts must include a data availability statement. This statement should provide the following information, where applicable:

- Accession codes, unique identifiers, or web links for publicly available datasets
- A description of any restrictions on data availability
- For clinical datasets or third party data, please ensure that the statement adheres to our policy

All data analyzed in this paper are available in raw form from their original authors. Specifically, the DLPFC dataset is accessible within the spatialLIBD package (http://spatial.libd.org/spatialLIBD). The MouseBrain dataset is collected from the 10x Genomics website (https://support.10xgenomics.com/spatial-gene-expression/datasets). Slide-seqV2 datasets are available at https://singlecell.broadinstitute.org/single_cell/study/SCP815/highly-sensitive-spatial-transcriptomics-at-near-cellular-resolution-with-slide-seqv2#study-summary. Slide-seq datasets are available at https://portals.broadinstitute.org/single_cell/study/slide-seq-study. The processed Stereo-seq data from mouse olfactory bulb tissue is accessible on https://github.com/JinmiaoChenLab/SEDR_analyses. The mouse visual cortex

STARmap data is accessible on https://www.dropbox.com/sh/f7ebheru1lbz91s/AADm6D54GSEFXB1feRy6OSASa/visual_1020/20180505_BY3_1kgenes?dl=0&subfolder_nav_tracking=1. The ISH images of the adult human brain are available at the Allen Human Brain Atlas (https://human.brain-map.org/).

Field-specific reporting

Please select the one below that is the best fit for your research. If you are not sure, read the appropriate sections before making your selection.

- [x] Life sciences
- [ ] Behavioural & social sciences
- [ ] Ecological, evolutionary & environmental sciences

For a reference copy of the document with all sections, see nature.com/documents/nr-reporting-summary-flat.pdf

Life sciences study design

All studies must disclose on these points even when the disclosure is negative.

Sample size	No sample size calculation was performed. All data used in this manuscript were taken from public resources and used to demonstrate the functionalities of STAGATE. The DLPFC dataset was determined because it have been manually annotated by experts, and the comparison of clustering accuracy is performed in this dataset. Moreover, we focus on the datasets from mouse hippocampus tissue and mouse olfactory bulb tissue due to our prior expertise on the aspects of Ammon's horn field and the laminar organization of mouse olfactory bulb. Specifically, we apply STAGATE to two datasets generated by Slide-seqV2 (mouse hippocampus tissue and mouse olfactory bulb tissue), one dataset generated by Stereo-seq (mouse olfactory bulb tissue), and one 3D ST dataset generated by Slide-seq (mouse hippocampus tissue). The Slide-seqV2 datasets and Stereo-seq dataset are used to show STAGATE's ability in identifying small spatial domain and complex spatial structures. And the 3D ST dataset is used to demonstrate the ability of STAGATE to identify 3D spatial domains. We use ST datasets generated from different technologies and at different spatial resolutions. Therefore, it is sufficient to demonstrate the functionalities of STAGATE
Data exclusions	We performed quality control of spatial resolved transcriptomics data based on the common used and pre-established criteria in this filed.
Replication	All Attempts at replication were successful and can be performed independently
Randomization	The allocation was random
Blinding	All results are based on published data which have been studied in their original publications. Therefore, blinding from investigators is not possible when we reanalyzed the data. Group allocation information was never provided to the computational algorithms.

Reporting for specific materials, systems and methods

We require information from authors about some types of materials, experimental systems and methods used in many studies. Here, indicate whether each material, system or method listed is relevant to your study. If you are not sure if a list item applies to your research, read the appropriate section before selecting a response.

Materials & experimental systems

n/a	Involved in the study	
[x]	[ ]	Antibodies
[x]	[ ]	Eukaryotic cell lines
[x]	[ ]	Palaeontology and archaeology
[x]	[ ]	Animals and other organisms
[x]	[ ]	Human research participants
[x]	[ ]	Clinical data
[x]	[ ]	Dual use research of concern

Methods

n/a	Involved in the study	
[x]	[ ]	ChIP-seq
[x]	[ ]	Flow cytometry
[x]	[ ]	MRI-based neuroimaging
